# Do Random Forest-Driven Climate Envelope Models Require Variable Selection? A Case Study on *Crustulina guttata* (Theridiidae: Araneae)

**DOI:** 10.3390/insects16020209

**Published:** 2025-02-14

**Authors:** Tae-Sung Kwon, Won Il Choi, Min-Jung Kim

**Affiliations:** 1 Alpha Insect Diversity Lab, Nowon, Seoul 01746, Republic of Korea; 2 Forest Entomology and Pathology Division, National Institute of Forest Science, Seoul 02455, Republic of Korea

**Keywords:** random forest, species distribution model, climate envelope model, variable selection, full model hypothesis, *Crustulina guttata*, multicollinearity

## Abstract

Climate Envelope Models (CEMs) commonly employ 19 bioclimatic variables to predict species distributions, yet selecting which variables to include remains a critical challenge. Although it seems logical to select ecologically relevant variables, the biological responses of many target species are poorly understood. Random Forest (RF), a popular method in CEMs, can effectively handle correlated and nonlinear variables. In light of these strengths, this study explores the full model hypothesis, which involves using all 19 bioclimatic variables in an RF model, using *Crustulina guttata* (Theridiidae: Araneae) as a test case. Four model variants—a simplified model with two variables, an ecologically selected model with seven variables, a statistically selected model with ten variables, and a full model with nineteen variables—were compared against a thousand randomly assembled models with matching variable counts. All models achieved high performance, though results varied based on the number of variables employed. Notably, the full model consistently produced stronger predictions than models with fewer variables. Moreover, specifying particular variables did not yield a significant advantage over random selections of equally sized sets, indicating that omitting variables may risk the loss of important information. Although the final model suggests that *C. guttata* may have dispersed beyond its native European range through artificial means, this study examined only a single species. Thus, caution is warranted in generalizing these findings, and additional research is needed to determine whether the full model hypothesis extends to other taxa and environmental contexts. In scenarios where ecological knowledge is limited, however, using all available variables in an RF model may preserve potentially significant predictors and enhance predictive accuracy.

## Simple Summary

The Climate Envelope Model (CEM) typically uses 19 bioclimatic variables to predict species distribution, but selecting ecological meaningful variables for target species is challenging. Random Forest (RM) models, which handle variable correlation, interaction, and nonlinearity well, were tested using an approach that includes all 19 variables. This was compared to three other model variants: a simplified model with two variables, a model with ecologically selected variables, and a model with statistically selected variables. The model using all variables generally performed better than those with fewer variables, and models with randomly selected variables often outperformed manually curated ones, showing the risks of losing important information during variable selection. The findings suggest that *Crustulina guttata* may have been artificially spread from Europe and highlight the advantages of using all available variables in RF models when the biological responses of a species are unclear. However, further research is certainlynecessary to confirm these results across other species and environmental contexts.

## 1. Introduction

Human-induced climate change and habitat destruction are profoundly altering species distributions worldwide, reshaping regional biota at an unprecedented rate [1,2,3]. In response to these shifts, Species Distribution Models (SDMs) have become invaluable tools for predicting changes in species distributions and broader ecological communities [4,5]. The integration of advanced statistical and machine learning techniques has significantly advanced the sophistication of SDMs [6]. Additionally, the expansion of accessible geographical distribution data, notably through the Global Biodiversity Information Facility (GBIF; https://www.gbif.org/), has greatly enhanced research capabilities within this field. SDMs are crucial for developing conservation strategies and formulating and testing hypotheses about geographic distributions, and are increasingly recognized for their ability to forecast the impacts of climate change on species distributions [4,6,7]. Furthermore, these models offer insights into the evolution of biological communities and diversity patterns in response to shifts in species distribution [8]. Beyond analyzing current and future trends, SDMs are used to infer the historical distributions of organisms using back-projected climates [5] and to predict the spread of diseases and invasive species, underscoring their versatile utility in tackling ecological and environmental challenges [9,10,11].

SDMs are grounded in ecological niche theory, which conceptualizes niches as multi-dimensional spaces shaped by environmental variables [12,13]. Among these, climatic niches are frequently used to predict the current and future distributions of species. These forecasts typically rely on climate change scenarios that utilize estimated current climatic niches. SDMs that employ climatic variables for distribution predictions are specifically referred to as Climate Envelope Models (CEMs) [5]. CEMs project shifts in species distributions due to climate change, commonly using a suite of 19 bioclimatic variables from WorldClim, which are derived from monthly temperature and precipitation data [7,14]. The selection and application of these variables are crucial to the accuracy of CEM forecasts [7,15,16].

In CEMs, variable selection is critical for accurately representing a species’ distribution and ensuring robust model performance [5,10]. When this process is not carried out appropriately, it can lead to the inclusion of highly correlated predictors. Such collinearity, especially in regression-based analyses, inflates parameter variance and can produce unstable predictions when models are applied to different regions or time periods with varying collinearity structures [17,18]. To address these issues, several statistical methods have been applied, including dimensionality reduction and threshold-based variable selection. Principal Component Analysis (PCA), for example, reduces the number of variables while preserving essential information, but it transforms original predictors into principal components and may limit biological interpretability [19]. Moreover, CEMs often employ a threshold-based approach (e.g., a Pearson correlation coefficient threshold of |r| > 0.7) to select variables [20,21]. These strategies can also help mitigate overfitting, which occurs when a model is excessively trained on a given dataset and does not generalize well to new data, as well as multicollinearity problems [22]. In addition, a simplified model with fewer variables can yield more intuitive interpretations for the target species.

However, there is a trade-off in deciding how many input variables to include: a model that is too simplified may lead to underfitting and fail to account for complex interactions among potentially confounding variables. Removing too many variables risks excluding key drivers of the species’ life history and overlooking potential interaction effects, particularly under high ecological uncertainty. Furthermore, choosing between correlated predictors often relies on the modeler’s subjective judgment, underscoring the difficulty of identifying the most ecologically relevant variables without a comprehensive understanding of the species’ specific climatic responses [23].

Despite these challenges, modern machine learning techniques are increasingly favored in CEMs for their effectiveness and resilience to collinearity issues [23,24]. Among these approaches, Random Forest (RF) has demonstrated superior performance over traditional regression-based methods in handling collinearity and improving model accuracy [10,18,25,26,27,28]. In particular, RF is noted for its tolerance to correlated variables [29], which leads to the hypothesis that including all available predictors without manual filtering (hereafter referred to as the “full model hypothesis”) could potentially enhance predictive accuracy in certain scenarios. Moreover, because RF constructs an ensemble of base learners from randomly selected subsets of input variables (bagging), it is considered relatively less prone to overfitting [30]. This characteristic may be especially pertinent when researchers lack a comprehensive understanding of how a target species responds to climatic factors, since using all variables might help avoid inadvertently discarding any that could be biologically significant.

This study aims to explore the validity of the full model hypothesis in an RF model by examining different variable selection strategies: those using ecologically informed selections, those based on statistically significant variables from correlated pairs, and a comprehensive model that incorporates the entire variable set. Additionally, the investigation includes an analysis of models that rely solely on key climatic variables, such as mean annual temperature and annual precipitation. This approach will provide nuanced insights into the efficacy of various variable selection strategies within CEMs, helping to identify potentially optimal configurations for accurate species distribution predictions.

For this research, *Crustulina guttata*, a spider species distributed across Europe and East Asia, was selected due to its distinct morphological characteristics, notably the unique white spots on its abdomen, which significantly reduce the risk of misidentification despite its small size. This species was relatively recently documented in South Korea and was first identified in 2001 [31,32]. This species is found in grasslands and sand dunes, with adults active from June to August [31], and it constructs small webs in vegetation close to the ground [33].

## 2. Materials and Methods

### 2.1. Occurrence Data

**Figure 1:**
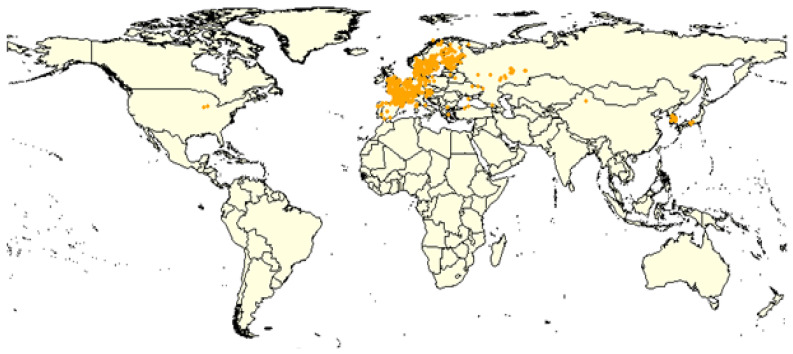
Geographic distribution of *Crustulina guttata*. Orange points represent the recorded locations of *C. guttata*, based on the GBIF database (accessed on 7 August 2023), occurrence records from a national survey by the National Institute of Forest Science (NIFOS), and additional data from other studies (see Table S1).

Occurrence (recorded sites) data for *C. guttata* were obtained from the GBIF (https://doi.org/10.15468/dl.6hkdej; accessed on 8 July 2023). Only records with valid coordinates were included, and any recorded prior to 2000 or located in marine areas were excluded. Additional distribution data were gathered from literature surveys [31,34,35] and museum specimens (https://species.nibr.go.kr/geo/html/index.do, accessed on 11 November 2022). In Korea, occurrence data also included records from national surveys by the National Institute of Forest Science (NIFOS), covering 200 sites from 2007 to 2009 [7] and 300 sites from 2017 to 2019 (unpublished data). These surveys employed pitfall traps, with detailed methods described in Kwon et al. [7]. After duplicate removal, these combined sources yielded 3846 presence points for *C. guttata*, as shown in Figure 1 and detailed in Table S1. This dataset was used as the primary occurrence dataset for distribution modeling. Additionally, 130 presence records were retrieved from iNaturalist (https://www.inaturalist.org; accessed on 18 January 2025). These records were kept separate from the main dataset and utilized exclusively for subsequent model evaluation and validation.

### 2.2. Covariates

In this study, we compared four models, each defined by a different set of bioclimatic variables, to evaluate how variable selection strategies influence species distribution predictions. The simplest approach is the two-factor model, which uses mean annual temperature (bio1) and annual precipitation (bio12). These two variables represent fundamental temperature and moisture gradients widely regarded as key drivers of species distributions.

Next, based on previous research, we selected seven bioclimatic variables that reflect critical elements of temperature extremes, seasonal fluctuations, and moisture levels essential for arthropod survival [7,11,13,16]. These seven variables include mean annual temperature (bio1), temperature seasonality (bio4), mean temperature of the warmest quarter (bio10), mean temperature of the coldest quarter (bio11), annual precipitation (bio12), precipitation of the wettest quarter (bio16), and precipitation of the driest quarter (bio17).

We then constructed a ten-factor model (statistically selected model) by examining correlations among 19 bioclimatic variables. For any pair with a correlation coefficient above 0.8, only the variable deemed more important, using the RandomForest package in R 4.1.2, was retained. Through this procedure, the variables selected were isothermality (bio3), temperature seasonality (bio4), maximum temperature of the warmest month (bio5), minimum temperature of the coldest month (bio6), mean temperature of the driest quarter (bio9), mean temperature of the coldest quarter (bio11), annual precipitation (bio12), precipitation seasonality (bio15), precipitation of the wettest quarter (bio16), and precipitation of the driest quarter (bio17). Notably, mean annual temperature (bio1) was excluded during this filtering, although five variables (bio4, bio11, bio12, bio16, and bio17) overlap with those in the ecologically selected model.

Finally, the full model uses all 19 bioclimatic variables without any filtering. This comprehensive approach allows us to test whether retaining all predictors, despite possible collinearity, can improve predictive performance. All bioclimatic layers used in this study were obtained at a 10 min spatial resolution. The combination of variables used in each model is summarized in Table 1.

### 2.3. CEM Modeling

During data preparation for the CEM, duplicate occurrence points within the same raster cell which is same size as the bioclimatic variables were identified and removed from the main dataset, which initially contained 3846 points. This process ensured that only 1 occurrence point was retained per cell, resulting in a total of 1024 presence points for CEM development.

The constructed distribution dataset potentially includes sampling bias [36]. Such bias can cause the CEM to learn sampling effort patterns during training, leading to misinterpretation of the model results. To address this, instead of using random background sampling, we adopted a strategy to sample background points from areas with a similar bias to the occurrence records [23]. Following the target-group sampling approach proposed by Kujala et al. [37], we collected distribution data for species within the same biological group (Order Araneae) as *C. guttata* from the GBIF and used these data to create a kernel density function. The kernel layer was generated using the *kde2d* function in the *MASS* package of R 4.1.2, matching the spatial resolution of the bioclimatic variables. Based on this density function, 1000 background points were sampled from areas located at least 2 decimal degrees away from the 1024 presence points [38]. The final dataset, consisting of both processed presence and pseudo-absence points, was randomly split into training (80%) and test (20%) datasets. Stratified data partitioning was applied to maintain the proportion of presence to pseudo-absence points in both subsets.

Through the use of the four variable compositions presented in Table 1, CEMs for *C. guttata* were constructed using the training data. Since the variables were predefined, these CEMs are referred to as specified models. The RF model was trained using the *randomForest* function in R 4.1.2, with default hyperparameter settings applied. Each variable composition was replicated ten times, resulting in ten models for each combination.

To further evaluate the effectiveness of our variable selection approach in improving model performance, we conducted an additional analysis in which the number of variables was fixed, but the variable combinations were randomized. These models are referred to as randomized models. In the randomized models, the number of variables matched those of the specified models in Table 1, but the variables themselves were randomly selected for training. For instance, when a randomized two-factor model was constructed, one variable was randomly selected from the temperature-related group (bio1–bio11) and another from the precipitation-related group (bio12–bio19), ensuring a balanced representation of climatic influences. In contrast, the specified two-factor model used predefined variables (bio1 and bio12) as shown in Table 1. A similar methodology was applied to the randomized seven-factor and ten-factor models. For the randomized seven-factor model, four variables related to temperature and three related to precipitation were randomly selected. Likewise, the randomized ten-factor model included six temperature-related variables and four precipitation-related variables, selected at random. The process of random variable selection, model construction, and evaluation was systematically replicated 1000 times for each variable combination to ensure consistency and reliability.

### 2.4. Model Evaluation

The performance of the RF models was evaluated using four metrics: the Area Under the Curve (AUC), True Skill Statistic (TSS), Boyce Index (BI), and Transferability. The AUC measures the model’s ability to distinguish presence points from background points, with values ranging from 0.5 (random prediction) to 1 (perfect prediction) [39]. The TSS evaluates the overall predictive accuracy of the model by accounting for both presence and background data [39]. It is calculated as the sum of sensitivity (true positive rate) and specificity (true negative rate) derived from the confusion matrix, minus 1. A TSS value of 1 indicates a perfect model. The BI evaluates whether the predicted probabilities of occurrence align with the observed frequency of presence points across the prediction gradient [40,41]. Higher BI values indicate better model performance in capturing the spatial patterns of presence data. The AUC and TSS were calculated using the *evaluate* function from the *dismo* package, and the BI was calculated using the *ecospat.boyce* function from the *ecospat* package in R 4.1.2.

Transferability was assessed as the model’s ability to generalize to an external dataset, using independent iNaturalist records. Specifically, Transferability was defined as the proportion of the 130 iNaturalist presence points correctly predicted as presence points by the model. For this, we applied the threshold that maximized both sensitivity and specificity for each model. Transferability scores ranged from 0 to 1, with higher values indicating better generalizability. All metrics were applied to both specified and randomized models, and all analyses were conducted in R 4.1.2.

The evaluation metrics for the specified models, which were replicated 10 times for each variable combination, were analyzed using the Kruskal–Wallis test at a 5% error rate. Post hoc comparisons were conducted using pairwise Wilcoxon tests with Bonferroni correction. For the randomized models, the evaluation metrics were compared with those of the specified models using identical numbers of variables. Specifically, each randomized model was compared to its corresponding specified model (e.g., the randomized two-factor model vs. the specified two-factor model) to assess whether variable selection improved model performance. This comparison was performed using Z-tests to determine whether the specified models consistently outperformed the randomized models.

### 2.5. Model Projection

To characterize the climatic requirements of *C. guttata*, the final RF model was constructed using the entire occurrence dataset, which included both training and test data, along with all 19 bioclimatic variables. The marginal effects of each variable in the full model were visualized using partial dependence plots, which illustrate the relationship between occurrence probability and climatic gradients. The final model, incorporating all variables and the complete occurrence data, was then projected onto geographic space to predict the climatic suitability of *C. guttata*.

## 3. Results

**Figure 2:**
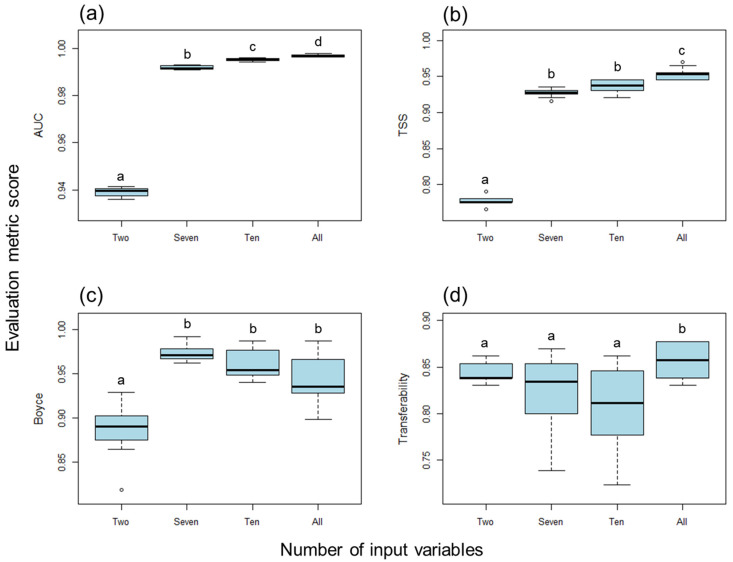
Distribution of (**a**) AUC, (**b**) TSS, (**c**) BI, and (**d**) transferability evaluation metrics for climate envelope model (CEM) using different specified combinations of bioclimatic variables: two-factor—bio1 and bio12; seven-factor—bio1, bio4, bio10, bio11, bio12, bio16, and bio17; ten-factor—bio3, bio4, bio5, bio6, bio9, bio11, bio12, bio15, bio16, and bio17. The selected input variables are detailed in Table 1. Different letters on the bars indicate significant differences between values (*p* < 0.05), as assessed by the Kruskal–Wallis test.

Figure 2 illustrates the distribution of evaluation metrics for the specified models, each constructed using different combinations of bioclimatic variables to describe the climatic envelope of *C. guttata*. Significant differences in model performance were observed depending on the combination of input variables, as indicated by the results of the Kruskal–Wallis rank sum test. For the AUC metric, there was a statistically significant difference across variable combinations (*χ*^2^ = 36.59, *df* = 3, *p* < 0.001). Similarly, significant differences were observed for the TSS (*χ*^2^ = 33.94, *df* = 3, *p* < 0.001) and BI (*χ*^2^ = 26.19, *df* = 3, *p* < 0.001). For Transferability, while differences were less pronounced, they remained statistically significant (*χ*^2^ = 9.79, *df* = 3, *p* = 0.02). While all specified models generally showed good performance, the full model using all 19 bioclimatic variables demonstrated superior performance for the AUC, TSS, and Transferability metrics (Figure 2). However, in terms of the BI, the full model performed slightly worse than the specified seven- and ten-factor models, though pairwise comparisons revealed no significant differences. The specified two-factor model, on the other hand, consistently showed significantly lower performance across all metrics.

**Figure 3:**
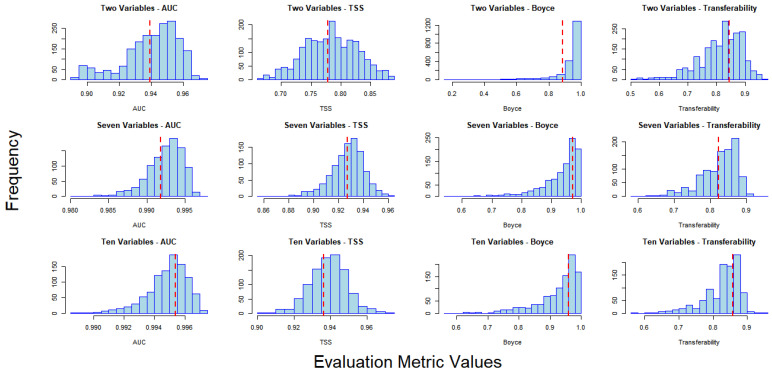
Histograms of evaluation metrics for randomized models (blue bars) compared to the mean values of specified models (red dashed lines). The rows represent different variable compositions: two-factor models (top row), seven-factor models (middle row), and ten-factor models (bottom row). The columns correspond to different evaluation metrics: AUC (leftmost column), TSS (second column), BI (third column), and Transferability (rightmost column).

When the specified models were compared to the randomized models with the same number of variables, no statistically significant differences in performance were observed (*p* > 0.05). Across all metrics, the specified two-factor, seven-factor, and ten-factor models did not outperform the upper range of distributions for the corresponding randomized models (Figure 3). The average performance metrics for the randomized models were as follows: The mean AUC values were 0.94 for the two-factor model, 0.99 for the seven-factor model, and 0.99 for the ten-factor model. Similarly, the TSS values averaged 0.78, 0.93, and 0.94 for the two-factor, seven-factor, and ten-factor models, respectively. For the BI, the averages were 0.93, 0.93, and 0.92, while Transferability values were 0.82, 0.83, and 0.83 for the same models. The randomized two-factor model exhibited lower AUC and TSS scores compared to the other models, but it showed minimal differences in the BI and Transferability. This pattern was also observed in the specified two-factor model.

**Figure 4:**
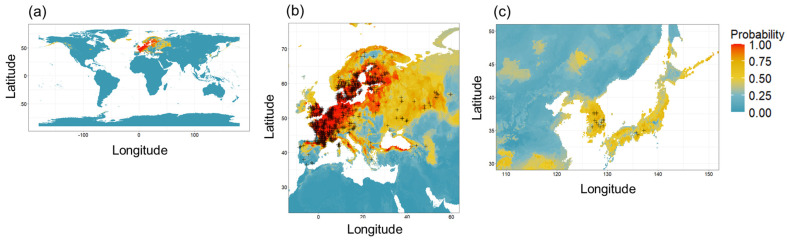
The predicted occurrence probability of *Crustulina guttata* based on the Random Forest model using all 19 bioclimatic variables. Predictions are displayed for (**a**) the global scale, (**b**) Europe, and (**c**) East Asia. Known presence points of *C. guttata* are indicated by cross marks (+).

The final full model, which utilized all 19 variables (i.e., the full model) and showed the highest performance, was projected onto geographic regions of Europe and East Asia (Figure 4). The predicted occurrence probability of the full model closely aligns with the known presence status of *C. guttata*. Western Europe and East Asia including the Korea peninsula and Japan were estimated to have higher climatic suitability.

**Figure 5:**
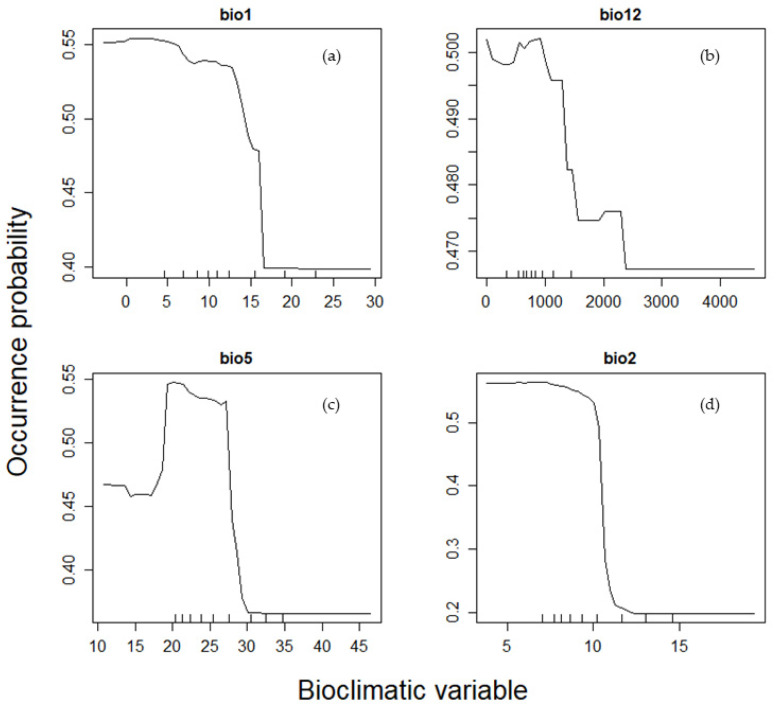
Partial dependence plots for four key bioclimatic variables from the full model using all 19 bioclimatic variables. The plots include two fundamental variables, mean annual temperature ((**a**): bio1) and annual precipitation ((**b**): bio12), as well as the two variables with the highest importance scores, maximum temperature of the warmest month ((**c**): bio5) and mean diurnal temperature range ((**d**): bio2).

Figure 5 displays the partial dependence plots for four key bioclimatic variables: bio1 (mean annual temperature), bio12 (annual precipitation), bio2 (mean diurnal temperature range), and bio5 (maximum temperature of the warmest month). Bio1 and bio12 represent fundamental climatic gradients, while bio2 and bio5 had high importance scores in the full model. The plots illustrate how the probability of *C*. *guttata* occurrence changes along the environmental gradients of these variables.

## 4. Discussion

Optimal variable selection in a CEM should ideally reflect the biological characteristics of the target species [13]. However, in this study, there was no distinct improvement in model performance when using specified input variables based on ecological and statistical considerations, while the full model (including all 19 variables) tended to show higher scores on certain evaluation metrics. Moreover, when the number of variables was fixed, the specified models did not significantly outperform the randomized models. This finding suggests that a model composed of variables deemed highly important does not necessarily outmatch models using randomly selected variables, likely due to limited knowledge of the target species’ biological responses or complex nonlinear interactions among multiple predictors. In other words, variable interactions or synergies may play a greater role in model performance than the individual variables themselves. Although the specified models generally performed well, they did not significantly surpass randomized models with the same number of variables. While an ecological rationale should be important in variable selection, randomly chosen variables may capture comparable predictive power, especially when the total number of variables is relatively large.

Previous studies have shown that machine learning algorithms, such as RF and MaxEnt, are relatively less affected by multicollinearity [18,28]. By contrast, algorithms like Boosted Regression Trees (BRTs) may benefit from reducing variable correlation, thereby minimizing overfitting [42], possibly due to the intrinsic mechanics of their ensemble structure [26]. The RF model, which uses a bagging technique to generate random subspaces for its individual decision trees, can mitigate the effects of multicollinearity by decreasing the dimensionality of the predictor space [43]. Pruning in these base learners, achieved by removing leaves, may further lessen correlation within these random subspaces [43,44]. Although extensive testing on additional species is necessary to generalize this hypothesis, our results support the robustness of the RF model against multicollinearity. In situations where ecological knowledge is limited, using all available variables may help preserve the diversity of random subspaces, potentially avoiding the loss of critical but unknown ecological information.

Despite the RF model’s known resilience against overfitting and our use of independent test data, the exceptionally high AUC values of the full model could still signal a potential overfitting risk. This concern might affect the model’s generalizability. Nevertheless, revalidation with independent iNaturalist data showed that the full model maintained slightly better performance. This outcome may reflect the inclusion of variables with currently unknown biological relevance or complex interactions among predictors. One advantage of correlative machine learning models like RF and MaxEnt is their ability to incorporate implicit effects of significant factors, such as host plant distributions, even when correlations with climate variables are low [37]. These models correlate occurrences and predictors directly, thus potentially capturing a realized niche that includes multiple underlying processes.

Our analysis provides only one example indicating that incorporating all available variables in a CEM can be beneficial, yet we caution against generalizing this finding. We focused on a single species and relied heavily on technical model evaluation metrics. Because we do not know the species’ true climatic suitability or actual distribution, these metrics may only reflect how well the model explains currently known distribution patterns. Furthermore, the optimal set of input variables may vary depending on the spatial scale of the study. In smaller or more localized regions, the range of climate variables is narrower, potentially altering collinearity structures and affecting model outcomes. In extreme cases, certain predictors could even become nearly perfectly correlated within a limited spatial extent. Finally, although correlative models that use bioclimatic variables can implicitly account for some interaction effects, they may be insufficient for species with strict habitat preferences. Under such circumstances, including additional environmental or ecological variables beyond the climate would be essential for capturing the species’ entire ecological niche. To further validate the full model hypothesis proposed in this study, future research should examine a variety of species under diverse conditions. In addition, the influence of RF hyperparameter tuning (such as the number of trees or the maximum depth of each tree) on model performance should be investigated to determine whether these parameters affect predictive accuracy in different ecological scenarios.

In the partial dependence plot of the full model, Bio1 and bio12 represent fundamental climatic gradients, while bio2 and bio5 had high importance scores in the full model. These plots illustrate how the probability of *C. guttata* occurrence changes along the environmental gradients of these variables. The response curve for bio1 suggests that *C. guttata* is vulnerable in regions with excessively high mean annual temperatures, indicating its preference for moderate thermal conditions. Similarly, the bio12 curve reveals that this species is less likely to thrive in areas with extremely high precipitation levels, suggesting an intolerance to overly wet environments. The response curve for bio5 exhibits a bell-shaped pattern, indicating that *C. guttata* has an optimal range for maximum temperature tolerance, beyond which its occurrence probability declines. Finally, the bio2 response suggests potential adaptation to maritime climates, implying that *C. guttata* is sensitive to diurnal temperature variability, a characteristic often associated with such regions.

The number of known *Crustulina* species is currently 17 (World Spider Catalog, accessed on 12 February 2024). In South Korea, two species, *C. guttata* and *C. sticta*, have been identified. This specific species composition is consistent in neighboring countries China and Japan [34,35], as well as in Ireland [33]. The uniformity in species composition across these regions may indicate that the current distribution of the genus *Crustulina* might have been shaped by artificial diffusion rather than natural dispersal. Although this study does not directly support the invasion possibility, it underscores the significant likelihood of such a scenario. The projected probability map suggests that the climatic envelope was not physically linked between East Asia and Europe, potentially considered its point of origin. These implications of climatic barriers on the spread of *Crustulina* further emphasize the role of environmental conditions in shaping distribution patterns. Moreover, *Crustulina* species, which prefer sandy soils, inhabit diverse environments including moors, heaths, coastal dunes, shingle habitats, woodland, and open grassland [33]. They build their nets in vegetation close to the ground, and adults are present during the summer [31]. These traits classify the species as habitat generalists, suggesting that their distribution is likely influenced more by climatic factors than by specific habitats. Therefore, it also appears quite plausible that *C. guttata* may have recently spread to new areas. The movement of species through artificial means, such as trade, is recognized as a significant threat to biodiversity conservation [45]. Although the focus often lies on pests that cause economic damage when species invasions are discussed, in reality, any species could potentially enter new areas through human activities. Therefore, it would be worthwhile to explore the invasion potential of a broader range of species using an accessible CEM.

This study experimentally tested CEMs for *C. guttata* using various numbers and combinations of bioclimatic variables. All models exhibited strong performance according to the AUC, although the results differed based on both the number and selection of variables. In particular, the best outcomes were observed when the full set of variables was employed, underscoring the potential information loss incurred by omitting potentially relevant predictors. Despite important limitations, including the focus on a single species and the evaluation of models primarily by climatic suitability differentiation, our findings suggest that using all available variables in an RF model can be advantageous, especially when biological information about the target species is limited. However, these results should not be overgeneralized. The optimal approach to variable selection may depend on factors such as the species’ ecology, spatial scale, and data availability. Moreover, when including non-climatic predictors such as topographic or land-use variables, careful selection or dimensionality reduction may be necessary to accommodate shifting multicollinearity structures across regions or time periods. Future research encompassing a broader range of species and ecological contexts, as well as investigations into RF hyperparameter tuning, could further clarify the conditions under which a full variable set offers the greatest benefits.

## Figures and Tables

**Table 1:** Description of bioclimatic variables and their selection for Climate Envelope Model (CEM). The check mark (√) indicates variables used in the model.

Variable	Code	Description	Model			
			Two	Seven	Ten	Full
Temperature	bio1	Annual Mean Temperature	√	√		√
	bio2	Mean Diurnal Range (Mean of monthly (max temp − min temp))				√
	bio3	Isothermality (BIO2/BIO7) (×100)			√	√
	bio4	Temperature Seasonality (standard deviation ×100)		√	√	√
	bio5	Max Temperature of Warmest Month			√	√
	bio6	Min Temperature of Coldest Month			√	√
	bio7	Temperature Annual Range (BIO5–BIO6)				√
	bio8	Mean Temperature of Wettest Quarter				√
	bio9	Mean Temperature of Driest Quarter			√	√
	bio10	Mean Temperature of Warmest Quarter		√		√
	bio11	Mean Temperature of Coldest Quarter		√	√	√
Precipitation	bio12	Annual Precipitation	√	√	√	√
	bio13	Precipitation of Wettest Month				√
	bio14	Precipitation of Driest Month				√
	bio15	Precipitation Seasonality (Coefficient of Variation)			√	√
	bio16	Precipitation of Wettest Quarter		√	√	√
	bio17	Precipitation of Driest Quarter		√	√	√
	bio18	Precipitation of Warmest Quarter				√
	bio19	Precipitation of Coldest Quarter				√

## Data Availability

The original contributions presented in this study are included in the article and Supplementary Materials. Further inquiries can be directed to the corresponding author.

## Supplementary Materials

The following supporting information can be downloaded at https://www.mdpi.com/article/10.3390/insects16020209/s1, Table S1: Occurrence records of *Crustulina guttata* from literatures and NIFOS investigations.
