# Supplementary material for: Do Random Forest-Driven Climate Envelope Models Require Variable Selection? A Case Study on Crustulina guttata (Theridiidae: Araneae)
Source: Insects. 2025 Feb 14;16(2):209. doi: 10.3390/insects16020209 (PMC11857067; doi:10.3390/insects16020209)
Supplement: Supplementary file 1 [file insects-16-00209-s001.zip › insects-3375184-supplementary.pdf]

insects

MDPI

Article

Do Random Forest-Driven Climate Envelope Models Require Variable Selection? A Case Study on *Crustulina guttata* (Theridiidae: Araneae)

Tae-Sung Kwon [1], Won Il Choi [2] and Min-Jung Kim [2,*]

Table S1. Occurrence records of *Crustulina guttata* from literatures and NIFOS investigations.

Species	Longitude	Latitude
Crustulina guttata	127.278	37.52175
Crustulina guttata	127.2831	37.461
Crustulina guttata	127.8998	37.58164
Crustulina guttata	127.4018	37.71183
Crustulina guttata	127.8973	37.58446
Crustulina guttata	129.0836	36.43992
Crustulina guttata	128.1917	35.91352
Crustulina guttata	128.5087	35.53371
Crustulina guttata	127.5621	36.55024
Crustulina guttata	126.9328	35.48504
Crustulina guttata	127.8989	36.18874
Crustulina guttata	129.1983	36.74039
Crustulina guttata	87.6271	43.79343
Crustulina guttata	137.1561	35.08256
Crustulina guttata	135.9182	34.3428
Crustulina guttata	135.6009	34.67929
